# Rubella Virus Triggers Type I Interferon Antiviral Response in Cultured Human Neural Cells: Involvement in the Control of Viral Gene Expression and Infectious Progeny Production

**DOI:** 10.3390/ijms23179799

**Published:** 2022-08-29

**Authors:** Sayuri Sakuragi, Huanan Liao, Kodai Yajima, Shigeyoshi Fujiwara, Hiroyuki Nakamura

**Affiliations:** 1 Department of Allergy and Clinical Immunology, National Research Institute for Child Health and Development, Tokyo 157-8535, Japan; 2 Department of Life Sciences, School of Agriculture, Meiji University, Kanagawa 214-8571, Japan

**Keywords:** rubella virus, innate immunity, interferon beta, type I interferon, interferon-stimulated gene, neural cell, MDA5, MAVS, TBK1, BX-795, congenital rubella syndrome

## Abstract

The type I interferon (IFN) response is one of the primary defense systems against various pathogens. Although rubella virus (RuV) infection is known to cause dysfunction of various organs and systems, including the central nervous system, little is known about how human neural cells evoke protective immunity against RuV infection, leading to controlling RuV replication. Using cultured human neural cells experimentally infected with RuV RA27/3 strain, we characterized the type I IFN immune response against the virus. RuV infected cultured human neural cell lines and induced IFN-β production, leading to the activation of signal transducer and activator of transcription 1 (STAT1) and the increased expression of IFN-stimulated genes (ISGs). Melanoma-differentiation-associated gene 5 (MDA5), one of the cytoplasmic retinoic acid-inducible gene I (RIG-I)-like receptors, is required for the RuV-triggered IFN-β mRNA induction in U373MG cells. We also showed that upregulation of RuV-triggered ISGs was attenuated by blocking IFN-α/β receptor subunit 2 (IFNAR2) using an IFNAR2-specific neutralizing antibody or by repressing mitochondrial antiviral signaling protein (MAVS) expression using MAVS-targeting short hairpin RNA (shRNA). Furthermore, treating RuV-infected cells with BX-795, a TANK-binding kinase 1 (TBK1)/I kappa B kinase ε (IKKε) inhibitor, robustly reduced STAT1 phosphorylation and expression of ISGs, enhancing viral gene expression and infectious virion production. Overall, our findings suggest that the RuV-triggered type I IFN-mediated antiviral response is essential in controlling RuV gene expression and viral replication in human neural cells.

## 1. Introduction

Innate immunity is one of the leading defense systems against various pathogens. Recognition of viral pathogen-associated molecular patterns (PAMPs) by pattern recognition receptors (PRRs) leads to the activation of innate immunity. Viral RNA PAMPs are recognized by retinoic acid-inducible gene I (RIG-I)-like receptors (RLRs), such as RIG-I [1], and melanoma differentiation-associated gene 5 (MDA5) [2] in the cytoplasm in various cell types [1,3,4,5,6,7]. Signaling mediated by RLRs is transduced by mitochondrial antiviral signaling protein (MAVS, also known as IPS-1, Cardif, or VISA), localized at the outer membrane of mitochondria [7,8,9,10]. Activation of MAVS leads to phosphorylation of the TANK-binding kinase 1 (TBK1)/I kappa B kinase ε (IKKε) complex and the I kappa B kinases α and β complexes (IKKα/IKKβ), resulting in the activation of interferon regulatory factor 3 (IRF3), IRF7, and nuclear factor kappa B (NF-κB). Activated IRF3/7 dimers and NF-κB translocate to the nucleus, triggering the production of type I interferons (IFNs) and various other humoral factors [11,12,13]. 

Type I IFNs activate the signaling pathway mediated by the Janus kinase/signal transducer and activator of transcription (JAK/STAT) by binding to their receptors (interferon alpha and beta receptor subunit 1 (IFNAR1) and interferon alpha and beta receptor subunit 2 (IFNAR2)). Phosphorylated STAT1 forms a heterodimer with phosphorylated STAT2, then recruits IRF9 to form IFN-stimulated gene factor 3 (ISGF3), which translocates to the nucleus, resulting in the activation of promoters of multiple IFN-stimulated genes (ISGs) through binding to IFN-stimulated responsive elements (ISREs) [14,15]. These ISGs include antiviral effectors, such as radical S-adenosyl methionine domain containing 2 (RSAD2)/viperin and interferon induced protein with tetratricopeptide repeats 1 (IFIT1)/ISG56, as well as PRRs, coordinating roles in controlling pathogen replication [16]. 

The rubella virus (RuV) belongs to the genus *Rubivirus* in the *Matonaviridae* family, including Guangdong Chinese water snake rubivirus [17,18], Fedallah virus [19], ruhugu virus, and rustrela virus [20]. RuV is an enveloped virus with a single-stranded positive-sense RNA genome. RuV has an RNA genome of approximately 10 kilobases, consisting of two open reading frames (ORFs). 5′-ORF encodes one precursor protein called P200, cleaved to the P150 and P90 proteins by RuV-encoded protease. The other precursor protein translated by sub-genomic RNA generated from the 3′ terminal of the RuV genome is cleaved to E1, E2, and a capsid, forming RuV progeny virions [21,22,23,24,25]. 

RuV infection in pregnant women, particularly in the first trimester, may result in intrauterine infection that causes damage in various fetal organs, including cataracts, heart disease, and hearing impairment, as well as neurologic disorders, including mental retardation, microcephaly, behavioral disorders, and meningoencephalitis; these fetal impairments comprise the congenital rubella syndrome (CRS) [22,23,24,26,27,28]. RuV infection may also cause rare neurological diseases, such as postinfectious encephalitis and progressive rubella panencephalitis [29,30,31,32,33]. Although the RuV vaccine efficiently prevents RuV infection and dramatically reduces CRS cases, the occurrence of multiple CRS cases is still a global health issue, particularly in regions with low vaccination coverage [21,34]. Notably, practical therapeutics for RuV-associated disease onset are not established. 

Although RuV infection potentially causes neurological symptoms with various brain imaging findings [35,36,37], little is known about how human neural cells evoke protective immunity against RuV infection, leading to controlling RuV replication. Using cultured human neural cells, we studied type I IFN-mediated innate immune responses against RuV infection. Our results suggest that RuV can commonly infect human neural cells and induce the IFN-β signaling pathway, which seems to play an important role in controlling RuV replication. 

## 2. Results

### 2.1. RuV Infects Cultured Human Neural Cells and Induces IFN-β Production

**Figure 1:**
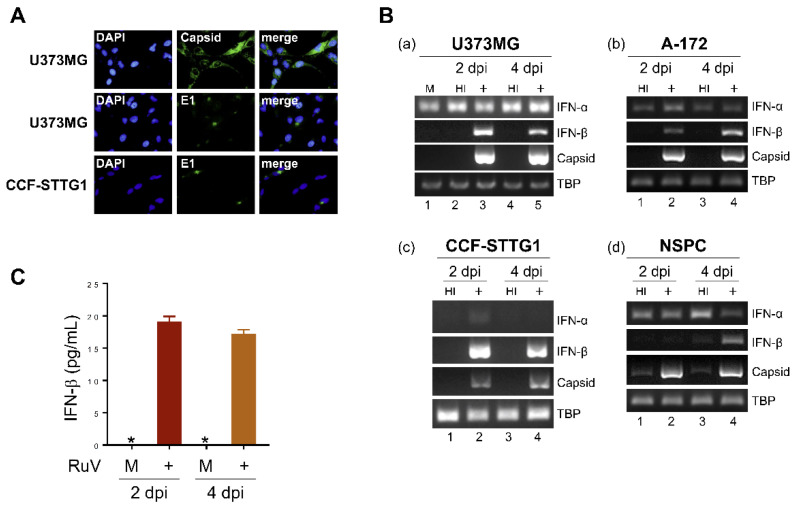
RuV infects human neural cell lines and induces IFN-β production. (**A**) Indirect immunofluorescence assay on U373MG cells and CCF-STTG1 cells infected with RuV RA27/3 strain. Infected cells at 3 days postinfection (dpi) were immunostained for RuV-encoded capsid or E1 proteins (green). DAPI was used for nuclear staining (blue). Images were acquired at a magnification of 40× objective lens. (**B**) Total RNAs extracted from neural cell lines U373MG (**a**), A-172 (**b**), CCF-STTG1 (**c**), and iPSC-derived NSPCs (**d**) that were uninfected (mock, M) or infected with RuV RA27/3 strain (+) or heat-inactivated RuV RA27/3 strain (HI) at 2 and 4 dpi (multiplicity of infection [MOI] of 1.8) were subjected to RT-PCR for the detection of IFN-α and IFN-β mRNAs and RuV RNAs encoding the capsid protein. TATA-box binding protein (TBP) mRNA was analyzed as a reference. The PCR products were analyzed by agarose gel electrophoresis. (**C**) Culture supernatants of U373MG cells that were uninfected (M) or infected with RuV RA27/3 strain (+) were collected at 2 and 4 dpi, and the concentration of IFN-β was measured by ELISA. Data are shown as means ± SEM of duplicate wells from one of two similar experiments. * indicates out-of-range.

To characterize type I IFN-mediated innate immune responses to RuV infection in human neural cells, we infected human neural cell lines (U373MG, CCF-STTG1, and A-172) and neural stem/progenitor cells (NSPCs) derived from human induced pluripotent stem cells (iPSCs) with the RuV RA27/3 strain. RuV-encoded capsid and E1 proteins were detected in the cytoplasm of U373MG and CCF-STTG1 cells by immunofluorescence assays (approximately 70% of U373MG cells expressed the capsid protein) (Figure 1A); RuV RNAs encoding the capsid protein were detected by conventional reverse transcription polymerase chain reaction (RT-PCR) (Figure 1B), indicating that the RuV RA27/3 strain can experimentally infect these neural cell lines. We next examined whether RuV infection induces type I IFN production. The mRNA expression of IFN-β, but not IFN-α, was upregulated in RuV-infected cells compared to uninfected (mock) or heat-inactivated-RuV-infected control cells (Figure 1B). In concordance with the RT-PCR data, increased amounts of IFN-β protein were detected in culture supernatants of RuV-infected U373MG cells, compared to mock-infected cells, by IFN-β-specific enzyme-linked immunosorbent assay (ELISA) (Figure 1C). These data suggest that cultured human neural cells are permissive to RuV infection, resulting in the expression of viral gene products and IFN-β production in vitro. 

### 2.2. RuV Infection Induces STAT1 Activation and ISGs Expression in Human Neural Cells

**Figure 2:**
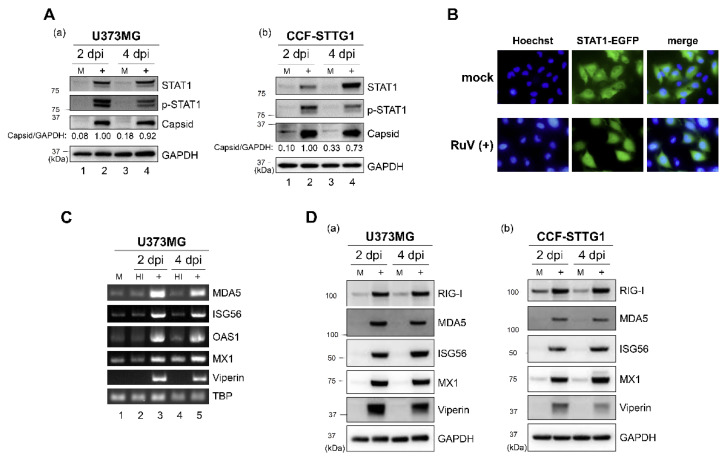
RuV infection induces STAT1 activation and ISG expression. (**A**) Protein lysates from U373MG cells (**a**) or CCF-STTG1 cells (**b**) that were uninfected (M) or infected with RuV RA27/3 strain (+) at 2 and 4 days postinfection (dpi) (MOI of 1.8) were analyzed by immunoblotting for STAT1, Tyr701-phosphorylated STAT1 (p-STAT1), and RuV-encoded capsid protein. GAPDH protein expression was analyzed as a reference. Densitometric analysis of the capsid protein was performed with NIH ImageJ software. Signal intensity of the capsid protein was normalized to that of GAPDH, then shown as a relative ratio of U373MG cells infected with RuV at 2 dpi. (**B**) STAT1-EGFP fusion protein (green) translocated to the nucleus was detected in RuV-infected U373MG-STAT1-EGFP cells at 2 dpi by fluorescence microscopy. Typical cells positive for nuclear localization of EGFP fusion protein are shown. Nuclei were counterstained with Hoechst 33342 (blue). Images were acquired at a magnification of 40× objective lens. (**C**) Total RNAs extracted from U373MG cells infected with RuV RA27/3 strain (+) or heat-inactivated RuV RA27/3 strain (HI) or uninfected (M) at 2 and 4 dpi were subjected to RT-PCR for the detection of expression of MDA5, ISG56, OAS1, MX1, and viperin mRNAs. TBP mRNA was analyzed as a reference. The PCR products were analyzed by agarose gel electrophoresis. (**D**) Protein lysates from U373MG cells (**a**) or CCF-STTG1 cells (**b**) that were uninfected (M) or infected with RuV RA27/3 strain (+) at 2 and 4 dpi (MOI of 1.8) were analyzed by immunoblotting for the protein expression of ISGs (RIG-I, MDA5, ISG56, MX1, and viperin). Glyceraldehyde-3-phosphate dehydrogenase (GAPDH) protein was analyzed as a reference.

As the IFN-β secreted from RuV-infected cells was presumed to activate downstream signaling through the IFN-α/β receptor, we examined whether RuV infection induces STAT1 activation. More phosphorylated-STAT1 (Tyr-701) protein was detected in RuV-infected U373MG and CCF-STTG1 cells compared to mock-infected cells (Figure 2A). We also analyzed the intracellular localization of STAT1 in RuV-infected cells using U373MG-STAT1-enhanced green fluorescent protein (EGFP) cells in which the STAT1-EGFP fusion protein was constitutively expressed. Nuclear localizing signals of STAT1-EGFP were detected in approximately 88% of cells after RuV infection, although being hardly detected in mock-infected cells (Figure 2B). These data indicate that RuV infection induced STAT1 activation in human neural cells. 

We next examined whether RuV infection could induce the expression of ISGs, as phosphorylated and nuclear localizing STAT1 would be expected to upregulate the expression of ISGs. Elevated mRNA expression of ISGs (interferon induced with helicase C domain 1 (IFIH1)/MDA5, IFIT1/ISG56, 2′-5′-oligoadenylate synthetase 1 (OAS1), MX dynamin like GTPase 1 (MX1), and RSAD2/viperin) was observed in RuV-infected U373MG cells, compared to mock-infected or heat-inactivated-RuV-infected control cells, by RT-PCR (Figure 2C). Immunoblotting also detected increased expression of ISG proteins (DExD/H-Box Helicase 58 (DDX58)/RIG-I, MDA5, ISG56, MX1, and viperin) in RuV-infected U373MG and CCF-STTG1 cells, compared to mock-infected cells (Figure 2D). These results, together with the observation of the RuV-triggered STAT1 activation, demonstrate that RuV infection increased the expression of ISGs in these cells. 

### 2.3. Anti-IFN-α/β Receptor Neutralizing Antibody Attenuates RuV-Induced Expression of ISGs

**Figure 3:**
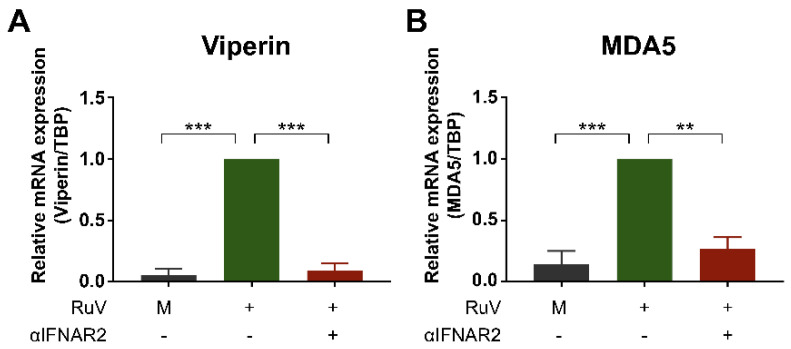
Anti-IFN-α/β receptor neutralizing antibody attenuates expression of RuV-induced ISGs. U373MG cells untreated (−) or pretreated (+) with an anti-IFNAR2 neutralizing antibody (αIFNAR2) for 18 h were infected with the RuV RA27/3 strain (+) at MOI of 1.8. The antibody-untreated U373MG cells uninfected with RuV (M) were used as a reference. Cells were harvested at 24 h postinfection and subjected to quantitative RT-PCR (qRT-PCR) to detect viperin mRNA (**A**) or MDA5 mRNA (**B**). Data are shown as the mean ± SEM (*n* = 3). Data were compared using one-way analysis of variance (one-way ANOVA) with Tukey’s test for multiple comparisons. **, *p* < 0.01; ***, *p* < 0.001.

To clarify whether the enhanced expression of ISGs observed after RuV infection would be mediated through the type I IFN-specific receptor, we attempted to attenuate the IFN-α/β receptor-mediated signaling using a neutralizing antibody against the IFNAR2. Pretreatment of U373MG cells with the neutralizing antibody reduced RuV-triggered expression of viperin mRNA (Figure 3A) and MDA5 mRNA (Figure 3B), suggesting that the type I IFN specific receptor-mediated IFN-β signaling plays an essential role in RuV-triggered expression of ISGs, such as viperin and MDA5, in U373MG cells. 

### 2.4. MDA5 Is Required for RuV-Triggered IFN-β Induction in U373MG Cells

**Figure 4:**
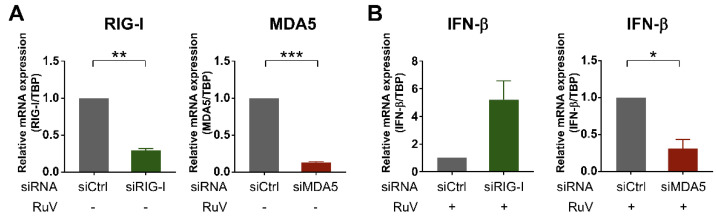
Silencing of MDA5 expression by siRNA reduced RuV-triggered IFN-β expression. (**A**) Effect of siRNA on the expression of MDA5 and RIG-I mRNAs. U373MG cells transfected with MDA5-targeting siRNA (siMDA5), RIG-I-targeting siRNA (siRIG-I), or a non-silencing control siRNA (siCtrl) at 9 nM were harvested at 48 h post-transfection. The mRNA expression levels of MDA5 and RIG-I were analyzed by qRT-PCR. Relative MDA5 or RIG-I mRNA levels in each sample were normalized to TBP and expressed as fold change relative to the corresponding control. Data are shown as the mean ± SEM (*n* = 3). Data were compared using a two-tailed Student *t* test with Welch’s correction. **, *p* < 0.01; ***, *p* < 0.001. (**B**) The expression of RuV-triggered IFN-β mRNA in siRNA-transfected U373MG cells. U373MG cells were infected with RuV RA27/3 strain at MOI of 1.8 at 48 h post-transfection of indicated siRNAs, harvested at 24 h postinfection, and subjected to qRT-PCR. Data are shown as the mean ± SEM (*n* = 3). Data were compared using a two-tailed Student *t* test with Welch’s correction. *, *p* < 0.05.

To examine whether cytoplasmic RLRs recognized RuV infection in human neural cells, we attempted to downregulate two cytoplasmic RLRs, RIG-I and MDA5, by transfecting small interfering RNA (siRNA) into U373MG cells. Small-interfering-RNA-mediated efficient knockdown of either RIG-I or MDA5 mRNA expression in U373MG cells was detected by quantitative RT-PCR (qRT-PCR) (Figure 4A). Next, siRNA-transfected U373MG cells were infected with the RuV RA27/3 strain at MOI of 1.8 at 48 h post-transfection of the siRNAs and harvested at 24 h postinfection and subjected to qRT-PCR. The induction of IFN-β mRNA triggered by RuV infection was clearly downregulated in siMDA5-transfected U373MG cells (Figure 4B, right), whereas no apparent downregulation of IFN-β mRNA was detected in siRIG-I-transfected U373MG cells (Figure 4B, left). These data indicate that MDA5 is likely required for the RuV-triggered induced expression of IFN-β mRNA in U373MG cells.

### 2.5. Knockdown of MAVS Attenuates Innate Immune Responses against RuV Infection

**Figure 5:**
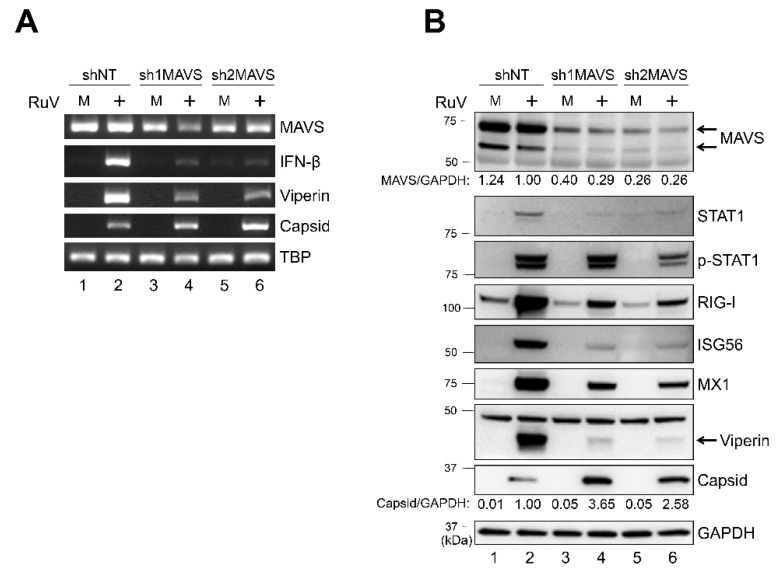
Short hairpin RNA-mediated MAVS knockdown attenuated the RuV-triggered IFN-β signaling pathway. (**A**) Total RNAs extracted from U373MG cells expressing MAVS-targeting shRNAs (sh1MAVS and sh2MAVS) or a non-targeting shRNA (shNT) that were uninfected (M) or infected with the RuV RA27/3 strain (+) at 2 dpi (MOI of 1.8) were subjected to RT-PCR for the detection of MAVS, IFN-β, and viperin mRNAs and RuV RNAs encoding the capsid protein. TBP mRNA was analyzed as a reference. The products were analyzed by agarose gel electrophoresis. (**B**) Protein lysates from shRNAs (sh1MAVS, sh2MAVS, and shNT)-expressing U373MG cells that were uninfected (M) or infected with the RuV RA27/3 strain (+) at 2 dpi (MOI of 1.8) were analyzed by immunoblotting for the protein expression of MAVS, STAT1, Tyr701-phosphorylated STAT1 (p-STAT1), ISGs (RIG-I, ISG56, MX1, and viperin), and RuV-encoded capsid proteins. GAPDH protein was analyzed as a reference. Densitometric analysis of MAVS and capsid proteins was performed with NIH ImageJ software. Signal intensity of those proteins was normalized to that of GAPDH, then shown as a relative ratio of shNT-expressing U373MG cells infected with the RuV RA27/3 strain.

To examine whether MAVS, which is critical for activating downstream signaling of MDA5, is involved in the RuV-induced type I IFN signaling, we established U373MG cells in which the MAVS expression was downregulated by MAVS-targeting short hairpin RNAs (shRNAs) (sh1MAVS and sh2MAVS). A lower level of MAVS expression was detected in shMAVS-expressing U373MG cells (sh1MAVS and sh2MAVS), compared to non-targeting shRNA (shNT)-expressing control cells, by RT-PCR (Figure 5A) and immunoblotting (Figure 5B). The mRNA expression of IFN-β and viperin induced by RuV infection was reduced in U373MG cells expressing sh1MAVS or sh2MAVS compared to the control cells (Figure 5A). Furthermore, the protein expression of STAT1, phosphorylated STAT1, and ISGs (RIG-I, ISG56, MX1, and viperin) were also reduced in sh1MAVS-expressing and sh2MAVS-expressing cells compared to the control cells (Figure 5B). These data suggest that MAVS is required for RuV-triggered IFN-β induction and the subsequent activation of STAT1 and expression of ISGs in neural cells. A higher expression of RuV-encoded capsid gene products was detected in shMAVS-expressing U373MG compared to shNT-expressing U373MG cells (Figure 5A,B), suggesting that the knockdown of MAVS also attenuated IFN-β-mediated repressive effects of RuV-encoded gene expression. 

### 2.6. BX-795, a TBK1/IKKε Inhibitor, Abrogates the RuV-Triggered Innate Immune Response and Augments the Production of Infectious Progeny Virions

**Figure 6:**
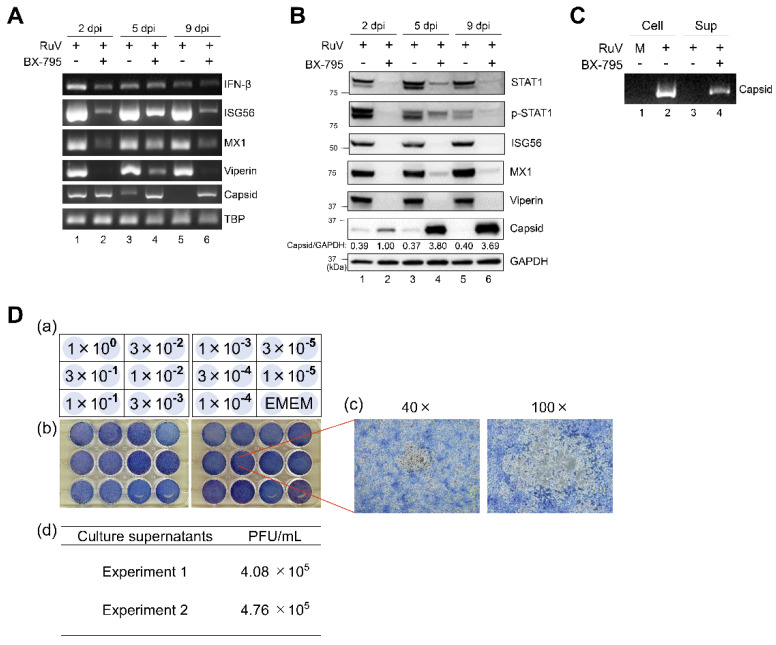
Effect of BX-795 on the RuV-triggered innate immune response and the production of infectious progeny virions. (**A**) Total RNAs extracted from RuV-infected U373MG cells (MOI of 1.8) pretreated with a TBK1/IKKε inhibitor BX-795 (10 μM) (+) or DMSO (−) for 3 h were harvested at the indicated times postinfection and subjected to RT-PCR for the detection of IFN-β, ISG56, MX1, and viperin mRNAs, and RuV RNAs encoding the capsid protein. TBP mRNA expression was analyzed as a reference. The products were analyzed by agarose gel electrophoresis. (**B**) Protein lysates from RuV-infected U373MG cells pretreated with BX-795 (+) or DMSO (−) were analyzed by immunoblotting for STAT1, Tyr701-phosphorylated STAT1 (p-STAT1), ISGs (ISG56, MX1, and viperin), and the RuV-encoded capsid protein. GAPDH protein expression was analyzed as a reference. Densitometric analysis of the capsid protein was performed with NIH ImageJ software. Signal intensity of the capsid was normalized to that of GAPDH, then shown as the relative ratio of BX-795-treated and RuV-infected U373MG cells at 2 dpi. (**C**) RuV virion RNAs extracted from supernatants (Sup) of RuV-infected U373MG cells treated with DMSO (−) or BX-795 (+) at 9 dpi were subjected to RT-PCR for the detection of RuV RNAs encoding the capsid protein. Cellular RNAs (Cell) extracted from uninfected (M) or RuV-infected U373MG cells (+) at 2 dpi were used as a reference. (**D**) Plaque assay to detect infectious RuV progeny virions in culture supernatants of RuV-infected U373MG cells treated with BX-795. Schematic representations of dilution series of the supernatants inoculated in duplicate (**a**) and photographs (**b**) of 12-well plates used in plaque assay of serially diluted culture supernatants. Enlarged photographs of plaques (40× and 100×) (**c**). Titers of infectious RuV progeny virions were measured using the supernatants of BX-795-treated U373MG cells collected at 9 days postinfection. Data were obtained in two independent experiments (**d**).

As MAVS-mediated type I IFN signaling derived from cytoplasmic sensors, such as MDA5, is known to progress through the activation of TBK1 and IKKε, we next examined the role of TBK1/IKKε in the RuV-triggered innate immune response. RuV-induced expression of IFN-β and ISG (ISG56, MX1, and viperin) mRNAs was attenuated by BX-795 treatment, compared to dimethyl sulfoxide (DMSO) treatment of RuV-infected U373MG cells (Figure 6A). Consistent with the RT-PCR data, immunoblotting showed the reduced expression of STAT1, phosphorylated STAT1, and ISG proteins, such as ISG56, MX1, and viperin. Of note, prolonged and increased expression of RuV-encoded capsid gene products was observed in BX-795-treated U373MG cells by RT-PCR and immunoblotting (Figure 6A,B). Then, we examined whether the downregulation of ISGs and the accumulation of RuV-encoded capsid gene products resulted in the enhanced production of infectious progeny virions. As expected, RuV virion RNAs were detected in culture supernatants of BX-795-treated U373MG cells infected with RuV by RT-PCR (Figure 6C, lane 4), although being hardly detectable in DMSO-treated cells (Figure 6C, lane 3). 

Furthermore, by plaque assay, we aimed to assess whether RuV virion RNAs originate from infectious progeny (Figure 6D). Our plaque assay indicated that virion RNAs detected in the supernatants of BX-795-treated U373MG cells were derived from progeny viruses characterized as infectious (Figure 6D). No plaque was found on any diluted supernatants of RuV-infected cells treated with DMSO that was performed as a reference (data not shown). These data indicate that TBK1/IKKε plays an essential role in RuV-triggered IFN-β induction, leading to STAT1 activation and ISG expression, resulting in controlling both viral gene expression and the production of infectious progeny virions. 

## 3. Discussion

The type I IFN response is an essential immune system component to defend against a broad range of pathogens. RuV infection causes profound effects on various organs and systems, including the central nervous system. The process of protective immunity of human neural cells against RuV infection leading to controlling RuV replication remains unclear. We characterized the type I IFN immune response against the virus using cultured human neural cells experimentally infected with the RuV RA27/3 strain. The results indicated that (1) several RuV-infected cultured human neural cell lines induced IFN-β production, leading to STAT1 activation and the upregulation of ISGs; (2) MDA5, one of the cytoplasmic RLRs, was required for the RuV-triggered IFN-β mRNA induction in U373MG cells; (3) RuV-triggered ISG upregulation was attenuated by blocking IFNAR2 or by repressing MAVS expression; and (4) BX-795 treatment of RuV-infected cells intensely attenuated STAT1 activation and ISG upregulation, leading to elevated production of infectious progeny virions. To our knowledge, the present study may be the first report regarding the characterization of IFN-β-mediated innate immune signaling against RuV infection in cultured human neural cells, although innate immune responses in human fetal and adult fibroblasts infected with the Cordoba wild-type strain of RuV were already reported [38]. 

In this study, the siRNA-mediated knockdown of MDA5, but not RIG-I, resulted in the reduction of the RuV-triggered expression of IFN-β mRNA, suggesting that MDA5 is required for sensing RuV infection and the following IFN-β induction in U373MG cells. MDA5 has been shown to preferentially recognize relatively high-molecular-weight poly (I:C) fragments [39,40], whereas RIG-I detects shorter dsRNA fragments. MDA5 senses picornaviruses [41], Sendai virus [42], mouse hepatitis virus [43], and murine norovirus 1 [44]. On the other hand, RIG-I preferentially recognizes the measles virus [45], Nipah virus [46], Ebola virus [47], and hepatitis C virus [48]. Although our findings suggest that MDA5 is likely to play an important role in recognizing RuV infection in human neural cells, we have not identified the exact PAMPs recognized by MDA5 in this study. Subgenomic fragments produced intermittently during RuV replication could be candidates for such MDA5 ligands. Furthermore, we cannot exclude the possibility that other PRRs, such as Toll-like receptors (TLRs) [49,50], also serve to recognize RuV infection in neural cells, as we focused on only the cytoplasmic PRRs RIG-I and MDA5 in this study. Further studies to clarify the RuV-sensing mechanism will aid in understanding more details in terms of anti-RuV innate immune signaling in neural cells. 

After interacting with the cytoplasmic viral RNAs, the sensor proteins, such as MDA5 and RIG-I, interact with MAVS to activate the downstream TBK1/IKKε [12,13], resulting in the induction of type I IFNs. Although shRNA-mediated downregulation of MAVS was somewhat limited, this downregulation weakened IFN-β induction, STAT1 phosphorylation, and ISG expression (Figure 5). Furthermore, the inhibition of another important mediator, TBK1/IKKε, by BX-795 demonstrated that BX-795 also efficiently attenuated both STAT1 activation and ISGs upregulation in RuV-infected U373MG cells (Figure 6). These data suggest that MAVS and TBK1/IKKε are likely to mediate RuV-triggered IFN-β expression and the following innate immune response in human neural cells. It is noteworthy that the treatment of RuV-infected U373MG cells with BX-795 caused remarkably enhanced RuV-encoded capsid expression and the production of infectious RuV progeny virions (Figure 6). These results highlight the importance of IFN-β-triggered innate immunity in controlling RuV replication. 

In this study, the expression of several ISGs was shown to be upregulated in RuV-infected cells (Figure 2). When the IFN-β-mediated signaling was blocked using an anti-IFNAR2 neutralizing antibody, RuV-induced expression of ISG mRNAs, such as viperin and MDA5, was downregulated (Figure 3), indicating that the IFN-β produced after RuV infection is indeed involved in the enhanced expression of at least some ISGs through the type I IFN receptor in U373MG cells. Although we focused on the role of type I IFN in controlling RuV infection in this study, emerging evidence suggests that a newly identified type III IFN-related immune system component also plays a crucial role in antiviral innate immune responses [51,52,53,54]. Further studies on antiviral responses mediated by other IFNs, including type III IFNs, will facilitate our comprehensive understanding of anti-RuV innate immune responses in human neural cells. In addition, as we analyzed the expression status of a limited number of ISGs, further comprehensive expression analyses may be required to identify ISGs whose expression is affected by RuV infection in more detail. Furthermore, our experiments were performed using the RuV RA27/3 vaccine strain in this study, because it has been well characterized and the conditions for its proper use are known. Future comparative studies using RuV wild-type strains will further clarify innate immune responses against RuV infection in human neural cells.

We have not yet elucidated which—and how—ISGs upregulated by RuV infection are currently involved in controlling RuV gene expression and progeny virion production. Of note, the inhibition of the IFN-β-mediated signaling pathway in RuV-infected cells by the expression of MAVS-targeting shRNA or the treatment with TBK1/IKKε-targeting BX-795 resulted in the augmentation of RuV-encoded capsid expression at both the transcriptional and protein levels (Figure 5 and Figure 6). These data suggest that the expression of RuV-encoded gene products may be regulated by ISGs, which repress viral gene expression at the transcriptional level, at least in part. Identification of ISGs contributing to the control of RuV-encoded gene expression and viral replication may serve to develop new strategies to control RuV pathogenesis. 

Taken together, our findings demonstrate that cultured human neural cells evoke type I IFN immune responses against RuV infection that are likely to play an essential role in controlling both RuV gene expression and the production of infectious progeny virions. More studies to clarify how innate immune responses against RuV infection link to neurological manifestations observed in RuV-infected individuals will contribute to our understanding of the RuV life cycle and may help design new therapeutic strategies to control RuV pathogenesis. 

## 4. Materials and Methods

### 4.1. Cells and Virus

A human glioblastoma cell line, A-172 (ATCC CRL-1620), and a human glioblastoma-astrocytoma cell line, U373MG (a gift from Dr. Naoki Inoue, Gifu Pharmaceutical University), were grown in Dulbecco’s modified Eagle’s medium (DMEM) (Nacalai Tesque, Kyoto, Japan) supplemented with 10% fetal bovine serum (FBS) (BioWest, Nuaillé, France), penicillin (200 U/mL, Nacalai Tesque), and streptomycin (200 μg/mL, Nacalai Tesque) at 37 °C in a 5% CO_2_ atmosphere. A human astrocytoma cell line, CCF-STTG1 (ATCC CRL-1718), was grown in RPMI-1640 medium (Fujifilm Wako Pure Chemical, Osaka, Japan) supplemented with 10% FBS and antibiotics. An African green monkey kidney cell line, Vero (ATCC CCL-81), was grown in Eagle’s minimum essential medium (EMEM) (Fujifilm Wako Pure Chemical) supplemented with 10% FBS and antibiotics. The Gibco episomal human induced pluripotent stem cell (iPSC) line (Thermo Fisher Scientific, Waltham, MA, USA) was grown in complete Essential 8 medium (Thermo Fisher Scientific) on vessels coated with vitronectin (Thermo Fisher Scientific). Neural stem/progenitor cells (NSPCs) derived from the iPSCs were obtained using PSC neural induction medium (Thermo Fisher Scientific) according to the manufacturer’s instructions. The RuV RA27/3 strain (ATCC VR-1359) was propagated in Vero cells. The virus stock solution containing RuV virions was added to Vero cells at 37 °C in a 5% CO_2_ atmosphere. At 2 days postinfection, culture supernatants were replaced with fresh EMEM supplemented with 10% FBS. At 8 days postinfection, culture supernatants were collected, centrifuged, filtered through a 0.45 µm PES filter system (Thermo Fisher Scientific, 165-0045), and stored at −80 °C in 5 mL aliquots. The titer of stock virus was determined on Vero cells by the plaque assay with methyl cellulose 4000 (Fujifilm Wako Pure Chemical) overlay and followed by staining with methylene blue (Sigma-Aldrich, St. Louis, MO, USA)solution as described in reference [55] with some modifications. Neural cells were infected with RuV RA27/3 strain at 37 °C for 3 h in a 5% CO_2_ atmosphere, and the virus inoculum was replaced with fresh medium. Cells were collected at the indicated time points postinfection for subsequent experiments. Heat-inactivated RuV was prepared by heating the virus stock solution at 65 °C for 3 h.

### 4.2. Reagents and Plasmids

The TBK1/IKKε inhibitor BX-795 was purchased from Sigma Aldrich. Wild-type STAT1 gene fused to an EGFP was cloned to pEBMulti-Hyg expression vector (Fujifilm Wako Pure Chemical). 

### 4.3. RNA Extraction and RT-PCR

Total RNA was extracted from cells using the ReliaPrep RNA Cell Miniprep System (Promega, Madison, WI, USA) according to the manufacturer’s protocol and quantified by the Qubit 3.0 system (Thermo Fisher Scientific) using the Qubit RNA BR assay kit (Thermo Fisher Scientific). Complementary DNA (cDNA) was synthesized from total RNA using PrimeScript IV 1st strand cDNA synthesis mix (Takara Bio, Otsu, Japan). Comparison of mRNA expression between samples was performed by either conventional RT-PCR or quantitative reverse transcription PCR (qRT-PCR). Conventional RT-PCR was performed in a total volume of 25 μL for 32 cycles using the Veriti thermal cycler (Thermo Fisher Scientific), and then the PCR products were analyzed by 1–2% agarose gel electrophoresis. Specific primer sets are described in Table S1. For quantitative reverse transcription-PCR (qRT-PCR), cDNA samples were amplified in Luna Universal Probe qPCR Master Mix (NEB, Ipswich, MA, USA) with TaqMan Gene Expression assay (Thermo Fisher Scientific) for interferon beta 1 (IFNB1, Hs01077958_s1), TATA box binding protein (TBP, Hs00427620_m1), DEXD/H-box helicase 58 (DDX58, Hs01061436_m1), interferon induced with helicase C domain 1 (IFIH1, Hs00223420_m1), and radical S-adenosyl methionine domain containing 2 (RSAD2, Hs00369813_m1). Each cDNA sample was amplified in triplicate with the StepOne Plus System (Thermo Fisher Scientific). Gene expression levels were quantitated by ΔΔCt analysis and normalized to TBP. 

### 4.4. Immunoblotting Assay

Cells were lysed in RIPA Buffer (Fujifilm Wako Pure Chemical) supplemented with protease inhibitors (cOmplete ULTRA tablets, Mini, EASYpack protease inhibitor cocktail, Roche, Basel, Switzerland) and centrifuged at 15,600× *g* at 4 °C for 15 min. The protein concentration of the supernatants was measured by comparison with 2 mg/mL albumin solution from bovine serum (Fujifilm Wako Pure Chemical) using the Protein Assay BCA Kit (Fujifilm Wako Pure Chemical) on a PICOSCOPE PAS-110 (Ushio Inc., Tokyo, Japan). The lysates were mixed with Laemmli SDS sample buffer with a reducing reagent (Nacalai Tesque), boiled at 95 °C for 5 min, and subjected to sodium dodecyl sulfate-polyacrylamide gel electrophoresis (SDS-PAGE). Protein samples were electrophoresed on 10–20% SuperSep Ace gels (Fujifilm Wako Pure Chemical) with running buffer (Fujifilm Wako Pure Chemical) and transferred onto PVDF membrane with the Trans-Blot Turbo RTA Mini PVDF Transfer Kit (Bio-Rad, Hercules, CA, USA). Membranes were blocked with Tris Buffered Saline with Tween 20 (TBS-T) Tablets (Takara Bio) dissolved in water containing 5% skim milk for immunoassay (Nacalai Tesque), incubated with a primary antibody overnight at 4 °C in TBS-T, visualized with species-specific horseradish peroxidase (HRP)-conjugated secondary antibodies, and developed using ImmunoStar Zeta (Fujifilm Wako Pure Chemical). Protein imaging was performed using the GE LAS4000 PhosphorImager (GE Healthcare Life Sciences, Pittsburgh, PA, USA) and quantified by ImageJ software (NIH, Bethesda, MD, USA). Each density measurement was normalized using the corresponding GAPDH level as a reference. 

The following primary antibodies were used for immunoblotting: anti-DDX58/RIG-I (1:1000, R&D Systems, Minneapolis, MN, USA, AF4859), anti-MX1 (1:1000, R&D Systems, AF7946), anti-MAVS (1:200, Santa Cruz Biotechnology, Dallas, TX, USA, sc-166583), anti-RSAD2/viperin (1:1000, Proteintech, Rosemont, IL, USA, 28089-1-AP), anti-IFIT1/ISG56 (1:1000, Proteintech, 23247-1-AP), anti-GAPDH conjugated with horseradish peroxidase (HRP) (1:10,000, Fujifilm Wako Pure Chemical, 015-25473), anti-IFIH1/MDA5 (1:500, Cell Signaling Technology, Beverly, MA, USA, 5321), anti-STAT1 (1:1000, Cell Signaling Technology, 9175), anti-phospho-STAT1 Tyr-701 (1:1000, Cell Signaling Technology, 7649), anti-RuV capsid (1:1000, Abcam, Cambridge, UK, ab34749), HRP-linked anti-rabbit IgG (1:10,000, Cytiva, Marlborough, MA, USA, NA934), and HRP-linked anti-mouse IgG (1:10,000, Cytiva, NA931). 

### 4.5. Indirect Immunofluorescence Assay

Cells were grown on 35 mm glass dishes (AGC Techno Glass, Shizuoka, Japan) and infected with RuV RA27/3 strain for 6 h, and the virus inoculum was replaced with a complete medium at 37 °C in a 5% CO_2_ atmosphere. At 3 days postinfection, the cells were fixed with 4% paraformaldehyde for 15 min, washed with phosphate-buffered saline without calcium or magnesium (PBS (−)), permeabilized with 1% Triton X-100 in PBS (−) for 5 min, and washed three times with PBS (−). Cells were incubated with anti-RuV capsid (1:250, MilliporeSigma, Burlington, MA, USA, MAB9251) and anti-RuV E1 (1:250, MilliporeSigma, MAB925) antibodies in PBS (−) containing 5% normal goat serum overnight at 4 °C and then incubated with Alexa Fluor 488 goat anti-mouse immunoglobulin G (IgG) (H+L) (1:250, Abcam) secondary antibody. Nuclei were stained with 4′,6-diamidino-2-phenylindole (DAPI, Dojindo Molecular Technologies, Kumamoto, Japan) or Hoechst 33342 (Dojindo Molecular Technologies). Images were captured using the Olympus IX71 system (Olympus, Tokyo, Japan). 

### 4.6. Enzyme-Linked Immunosorbent Assay (ELISA)

Cell-free culture supernatants were collected from RuV-infected cells at the indicated times after RuV infection. According to the manufacturer’s instructions, the amount of human IFN-β was quantified using the VeriKine human IFN-β ELISA kit (PBL Assay Science, Piscataway, NJ, USA, 41410). The absorbance of test samples was measured using the Wallac 1420 ARVOsx multi-label counter (Perkin Elmer, Waltham, MA, USA) and converted to picograms per milliliter using a standard curve generated by serially diluting the standard in the sample plate. Indicated data are representative of two experiments. 

### 4.7. Interferon α/β Receptor Neutralization Assay

U373MG cells were treated with an anti-interferon α/β receptor chain 2 monoclonal antibody (MilliporeSigma, MAB1155, clone MMHAR-2) at 4 μg/mL for 18 h before RuV RA27/3 strain infection. Cells were collected at 24 h postinfection and subjected to qRT-PCR. 

### 4.8. Lentiviral shRNA Transduction

Short hairpin RNAs (shRNAs) were expressed from pLKO.2 lentiviral vectors (Sigma-Aldrich). To generate lentiviral particles, the plasmid constructs were cotransfected with MISSION Lentiviral Packaging Mix (Sigma-Aldrich, SHP001) into Lenti-X 293T cells (Takara Bio). The MISSION lentiviral shRNA clones targeting MAVS transcripts (Sigma-Aldrich, TRCN0000236031 and TRCN0000236032) were transduced to U373MG cells. A non-target control shRNA (Sigma-Aldrich, SHC002) was used as a reference. U373MG cells infected with shRNA-expressing lentivirus were treated with 27 μg/mL of puromycin (InvivoGen, San Diego, CA, USA) for 3 weeks. 

### 4.9. BX-795 Treatment

U373MG cells were treated with 10 μM of BX-795 (Sigma-Aldrich) for 3 h before RuV RA27/3 strain infection. U373MG cells treated with DMSO (Sigma-Aldrich) were used as a reference. Culture supernatants were replaced with DMEM supplemented with 10 μM of BX-795, 10% FBS, and antibiotics at 2 and 5 days postinfection. For reference, culture supernatants were changed with DMEM supplemented with an equal volume of DMSO, 10% FBS, and antibiotics at the exact timing with BX-795. Cells were collected at the indicated days postinfection of RuV and subjected to RT-PCR and immunoblotting. 

For the detection of virion RNAs in culture supernatants of RuV-infected U373MG cells treated with BX-795 or DMSO, culture supernatants were centrifuged and filtered through a 0.22 μm Millex-GV PVDF syringe filter (MilliporeSigma, SLGV033RS). Virion RNAs were extracted using the QIAamp MinElute Virus Spin Kit (Qiagen, Hilden, Germany) according to the manufacturer’s instructions, then reverse-transcribed to cDNAs using PrimeScrip IV 1st strand cDNA Synthesis Mix (Takara Bio). RT-PCR was performed with the same volume of cDNA using an RuV-capsid primer set, and the products were analyzed by agarose gel electrophoresis. 

For plaque assays, culture supernatants of RuV-infected U373MG cells treated with BX-795 or DMSO were collected at 9 days postinfection, centrifuged, and filtered through a 0.22 μm Millex-GV PVDF syringe filter, and then serially diluted with EMEM. Vero cells were inoculated with the diluted culture supernatants, then fixed with formalin and stained with methylene blue solution at 10 or 11 days postinfection. The plaque numbers were calculated with values of PFU/mL. 

### 4.10. Small Interfering RNA Transfection

U373MG cells were seeded in 6 cm dishes (1 × 10^5^ cells per dish) 3 days before transfection and were transfected with 9 nM of small interfering RNA (siRNA) targeting RIG-I (Santa Cruz Biotechnology, sc-61480) or MDA5 (Silencer select predesigned siRNA, s34498, Thermo Fisher Scientific) using Lipofectamine RNAiMAX reagent (Thermo Fisher Scientific). Control siRNAs were also purchased from Santa Cruz Biotechnology (sc-37007) and Thermo Fisher Scientific (4390843). The cells were harvested at 48 h post-transfection and subjected to qRT-PCR. The cells were infected with the RuV RA27/3 strain at 48 h post-transfection of siRNAs, then harvested at 24 h postinfection and subjected to qRT-PCR. 

### 4.11. Statistical Analysis

Data are shown as mean values with standard error of the mean (SEM). Differences between experimental groups were determined by a two-tailed Student *t* test with Welch’s correction or one-way analysis of variance (one way ANOVA) with Tukey’s test for multiple comparisons using GraphPad Prism 7 software (GraphPad Software, San Diego, CA, USA). A *p* value less than 0.05 was considered statistically significant. 

## Data Availability

The data generated during the current study are available upon reasonable request.

## Supplementary Materials

The supporting information can be downloaded at: https://www.mdpi.com/article/10.3390/ijms23179799/s1.
