# Supplementary material for: Rubella Virus Triggers Type I Interferon Antiviral Response in Cultured Human Neural Cells: Involvement in the Control of Viral Gene Expression and Infectious Progeny Production
Source: Int J Mol Sci. 2022 Aug 29;23(17):9799. doi: 10.3390/ijms23179799 (PMC9456041; doi:10.3390/ijms23179799)
Supplement: Supplementary file 1 [file ijms-23-09799-s001.zip › ijms-1858969-supplementary.pdf]

Supplementary Table S1

Title: Rubella Virus Triggers Type I Interferon Antiviral Response in Cultured Human Neural Cells: Involvement in the Control of Viral Gene Expression and Infectious Progeny Production

Authors: Sayuri Sakuragi, Huanan Liao, Kodai Yajima, Shigeyoshi Fujiwara, and Hiroyuki Nakamura

Table S1: Primers used for RT-PCR

Gene	Forward primer (5'-3')	Reverse primer (5'-3')
IFN-α (consensus)	AGAATCTCTCCTTTCTCCTG	TCTGACAACCTCCCAGGCAC
IFN-β	ATGACCAACAAGTGTCTCCTCC	TCAGTTTCGGAGGTAACCTGTAAG
ISG56	GATGACGATGAAATGCCTGA	GCCCGCTCATAGTACTCCAG
MAVS	CCTACCACCTTGATGCCTGT	AAAGGTGCCCTCGGACTTAT
MDA5	TGCTCACAGTGGTTCAGGAG	TAAGCCTTTGTGCACCATCA
MX1	GCTACACACCGTGACGGATA	TTCAGGAGCCAGCTGTAGGT
OAS1	CTATCTCTTGCCAGACACGTG	CTCAGCCTCTTGTGCCAGCTG
RV capsid	GCTTCTACTACCCCCATCACC	GGCGCGCGCGGTGCCAACGGCGA
TBP	TTCGGAGAGTTCTGGGATTGTA	TGGACTGTTCTTCACTCTTGGC
Viperin	GTGAGCAATGGAAGCCTGAT	TCCTTCCGTCCCTTTCTACA
